# An early diagnosis is not the same as a timely diagnosis of Parkinson's disease

**DOI:** 10.12688/f1000research.14528.1

**Published:** 2018-07-18

**Authors:** Richard Nathaniel Rees, Anita Prema Acharya, Anette Schrag, Alastair John Noyce

**Affiliations:** 1 Department of Clinical Neuroscience, Institute of Neurology, UCL Hampstead Campus, London, UK; 2 Barts and the London School of Medicine and Dentistry, Queen Mary University of London, London, UK; 3 Preventive Neurology Unit, Wolfson Institute of Preventive Medicine, Queen Mary University of London, London, UK; 4 Department of Molecular Neuroscience, UCL Institute of Neurology, London, UK

**Keywords:** Parkinson's disease, neurodegeneration, prodromal, disease modifying therapy, ethics, personalized medicine

## Abstract

Parkinson’s disease is a common neurodegenerative condition that has significant costs to the individual patient and to society. The pathology starts up to a decade before symptoms are severe enough to allow a diagnosis using current criteria. Although the search for disease-modifying treatment continues, it is vital to understand what the right time is for diagnosis. Diagnosis of Parkinson’s disease is based on the classic clinical criteria, but the presence of other clinical features and disease biomarkers may allow earlier diagnosis, at least in a research setting. In this review, we identify the benefits of an early diagnosis, including before the classic clinical features occur. However, picking the right point for a “timely” diagnosis will vary depending on the preferences of the individual patient, efficacy (or existence) of disease-modifying treatment, and the ability for health systems to provide support and management for individuals at every stage of the disease. Good evidence for the quality-of-life benefits of existing symptomatic treatment supports the argument for earlier diagnosis at a time when symptoms are already present. This argument would be significantly bolstered by the development of disease-modifying treatments. Benefits of early diagnosis and treatment would affect not only the individual (and their families) but also the wider society and the research community. Ultimately, however, shared decision-making and the principles of autonomy, beneficence, and non-maleficence will need to be applied on an individual basis when considering a “timely” diagnosis.

Parkinson’s disease (PD) is an age-related neurodegenerative condition with a current prevalence of 41 out of 100,000 in people who are 40 to 49 years old, increasing to 1,607 out of 100,000 in those over the age of 80 years ^1– 3^. Global data indicate that PD will become a pandemic: prevalence more than doubled between 1990 and 2015, and PD now affects 6.2 million individuals. Applying recent trends to global population forecasts yields estimates of 12.9 to 14.2 million by 2040 ^1, 2, 4^. Although it has often been said that people with PD do not have a shortened life span, there is evidence to the contrary ^5, 6^, and “healthy life” is substantially shortened for many patients. Premature death is even more apparent in PD patients with dementia ^7^.

PD is widely recognized as the classic form of “the shaking palsy” or “paralysis agitans” first described by James Parkinson 200 years ago ^8^. The hallmark signs are bradykinesia (which describes decrement in a repetitive movement), rigidity, and tremor. Whereas these are the key motor features necessary for diagnosis, there are a host of non-motor symptoms which often emerge before the point of diagnosis. These non-motor symptoms convey a significant burden on the individual and their caregivers ^9^. They include pain, autonomic features (such as constipation, hypotension, and erectile dysfunction), psychiatric disturbance (such as memory problems, affective disorders, and apathy), and other features, including fatigue, rapid eye movement (REM) sleep behavior disorder (RBD), smell loss, and hypersalivation ^9, 10^.

The motor symptoms are caused by destruction of dopaminergic neurons, which occurs primarily in the substantia nigra ^11^. As with other neurodegenerative diseases, an aberrant form of a naturally occurring protein—in this case, α-synuclein—is implicated in the pathological cascade. Protein aggregation and neuronal death occur long before overt clinical features manifest ^12– 16^. At the point of diagnosis, there has been a 50 to 70% reduction in striatal dopamine and 30 to 50% of dopaminergic neurons have been lost ^17, 18^. Intervention before neuronal loss is advanced, and slowing of the disease process are major priorities for PD research.

To intervene earlier, it must be possible to identify people before they would usually receive a diagnosis. However, it is important to be clear about the difference between an “early” and a “timely” diagnosis. From a scientific perspective, “early” is easy to comprehend within the framework of disease pathology and its manifestations. Most neurodegenerative processes follow a pattern of progression from the nascent or pre-diagnostic phase through categorical disease stages and ultimately death. This perspective is objective and necessary for the mapping out of relationships, processes, and treatments. Following on from progress made in infectious diseases and cancer, the axiom that earlier detection is better is hard to refute when there are drugs available that can change the underlying disease process. However, “timely” does not necessarily fit with this approach and may mean different things to different patients and indeed to society. We have moved further from the paternalistic doctor–patient relationship of previous generations and more toward a person-centered, individualized approach to health care. “Timely” puts an approach to diagnosis within the multiple spectra of the individual’s priorities and recognizes both the potential advantages and the disadvantages of an earlier diagnosis. These arguments, many of which hold true for PD, have been summarized in the context of dementia by Dhedhi *et al*. ^19^ ( Figure 1).

**Figure 1.**
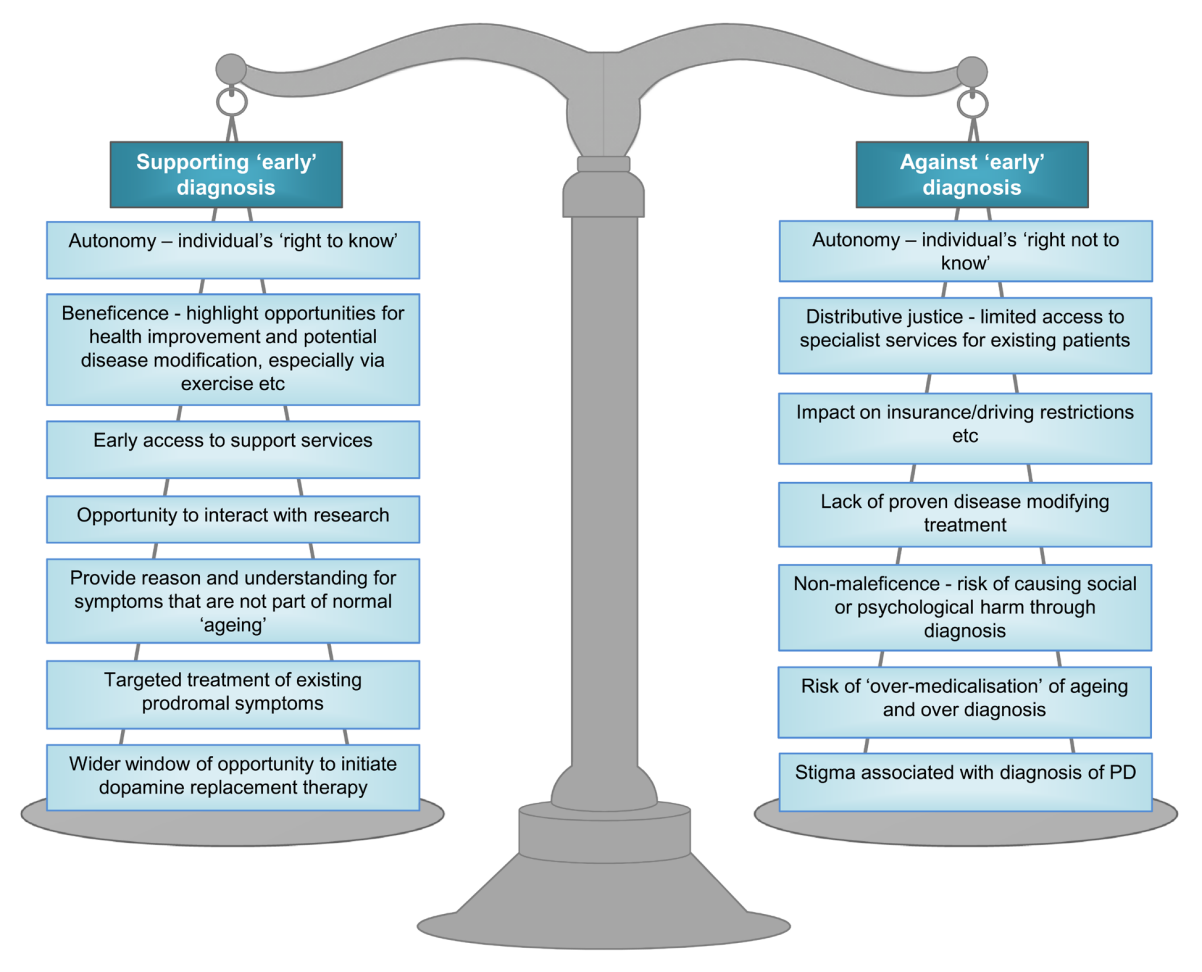
Arguments for and against “early” diagnosis. The ability to detect disease earlier is becoming more reliable, although no gold standard exists for a definitive diagnosis in life. However, as with all interventions in medicine, there are risks and benefits to an “early” diagnosis of Parkinson’s disease (PD). This figure summarizes the arguments. See Dhedhi *et al*. for a full discourse of these arguments relating to Alzheimer’s disease ^19^.

## Current approach to diagnosis

The “gold standard” for the diagnosis of PD is postmortem pathological examination of the brain showing α-synuclein-positive inclusions in neuronal cells that have not died. The clinical diagnosis should be made by those with experience in PD and it is accurate about 85% of the time ^20^. The most widely used diagnostic criteria for research are the United Kingdom Parkinson’s Disease Society Brain Bank Criteria, a three-step process that consists of confirming the presence of a Parkinsonian syndrome, ensuring the absence of any exclusion criteria, and establishing the presence of supportive criteria ^21^. It is an iterative process, and although a diagnosis of PD can often be made at the first consultation, it is a diagnosis that requires periodic review to ensure that it remains the best explanation.

Given the fallibility of clinical diagnosis, revised methods of diagnosis and the search for biomarkers continue. The International Parkinson and Movement Disorders Society (MDS) has recently proposed an update to the clinical criteria for PD ^22^. This allows a two-level diagnosis of “clinically established PD” and “clinically probable PD”. It takes an algorithmic approach to establishing the presence of a parkinsonian syndrome and then checking for “supportive”, “red flag”, and “absolute exclusion” criteria. These new criteria have the benefit of bringing attention to many of the non-motor symptoms, but whether they result in higher diagnostic accuracy overall remains to be determined. Interestingly, despite huge efforts, biomarkers—clinical, genetic, biochemical, or imaging (for a summary, see 23)—still play essentially no role, and even functional neuro-imaging (that is, dopamine transporter single-photon emission computed tomography [SPECT]) is only mentioned in the new criteria as an absolute exclusion when normal. National guidelines in the UK also do not recommend any biomarkers for routine clinical use, explicitly recommending that magnetic resonance imaging not be used to confirm the diagnosis outside of the research setting ^24^.

## Approaches to “early” diagnosis

There has been growing attention to the pre-diagnostic phase of PD, and several studies have been initiated to better characterize this using a variety of risk factors and markers and prodromal features ^25– 27^. The Parkinson’s Associated Risk Study (PARS) is a multicenter US study comparing older adults with and without hyposmia and conducts annual physical assessments and two yearly dopamine transporter SPECT scans ^28^. The PRIPS (Prospective Validation of Risk Factors for the Development of Parkinsonian Syndromes) study, in three European countries, compares risk factors, physical examination, smell, and transcranial sonography in 1,847 adults older than 50 years ^29^. The Tübingen Evaluation of Risk Factors for Early Detection of Neurodegeneration (TREND) cohort has followed nearly 700 individuals (aged 50 to 85) enriched for possible pre-diagnostic symptoms. Participants were enrolled if they had at least one of the following symptoms: depression, reduced smell, or RBD. Biannual assessments included motor and neuropsychological examinations, smell, quantitative gait and balance assessments, and transcranial sonography ^30^. The Parkinson’s Progression Markers Initiative (PPMI) is a multinational cohort with recruitment in North America, Europe, Israel, and Australia. In addition to a longitudinal study of 423 individuals with established PD, there is a control cohort of 196 individuals (aged over 30 years) with neither PD nor a first-degree relative with PD and a further 65 individuals in a specific “at-risk” cohort with RBD or hyposmia. The investigators are continuing to recruit individuals with and without PD who carry mutations in moderate- to high-risk genes ( *LRRK2*, *GBA*, or *SNCA*) ^31^. In the UK, the PREDICT-PD study, which has been running since 2011, has followed more than 1,300 older adults (aged from 60 to 80 at entry) without a diagnosis of PD or other neurological disease. Annual online assessments track motor and non-motor symptoms through validated, evidence-based questionnaires, a tapping test for motor slowing ^32^, and objective smell testing ^33, 34^. Although the methodologies vary, there is a clear indication that it is possible (and feasible) to detect people with strong evidence of “pre-diagnostic” PD through epidemiological, clinical, imaging, and other risk markers ^28, 35– 38^. As many of these cohorts mature, the numbers of “high-risk” individuals “converting” to established PD provide proof of concept and will help to establish the optimum approach to “early” detection. But whether this is “timely” remains a point of contention.

Separately, special mention should be given to the fact that a strong family history of PD or a genetically related condition could significantly alter an individual’s perspective on early versus timely diagnosis, but there also exists the opportunity for entry into genetically targeted disease-modifying treatment (DMT) trials. Gaucher’s disease (GD) is a lysosomal storage disease that is caused by mutations in the glucocerebrosidase gene ( *GBA*). It has been recognized that individuals with type 1 GD have a significantly increased risk of PD, and genetic testing of unselected PD cohorts reveals *GBA* mutations in up to 10% (and is significantly higher in some populations, such as Ashkenazi Jewish PD cohorts) ^39– 42^. In some populations, particularly Ashkenazi Jewish and Berber Arab populations, mutations in the leucine-rich repeat kinase 2 ( *LRRK2*) gene are found in a significant proportion of individuals with PD, and the *LRRK2* prodrome and established PD phenotypes, as well as the pathology, may differ from “idiopathic” PD ^43– 48^.

In addition to publishing the revised clinical criteria for a diagnosis of PD, the MDS has published criteria for prodromal PD for use as a research tool ^26^. These calculate a combined likelihood ratio for an individual using risk factors (age, sex, occupational toxin exposure, smoking and caffeine use, family history, and nigral hyperechogenicity using transcranial sonography) and prodromal features (RBD, abnormal dopaminergic imaging, quantitative motor testing, hyposmia, constipation, hypotension, erectile and urinary dysfunction, and depression/anxiety disorders). The cutoff for “probable prodromal PD” is at least 80% using the criteria and this has now been validated in several studies ^49– 51^.

## When is the most “timely” diagnosis for an individual?

**Figure 2.**
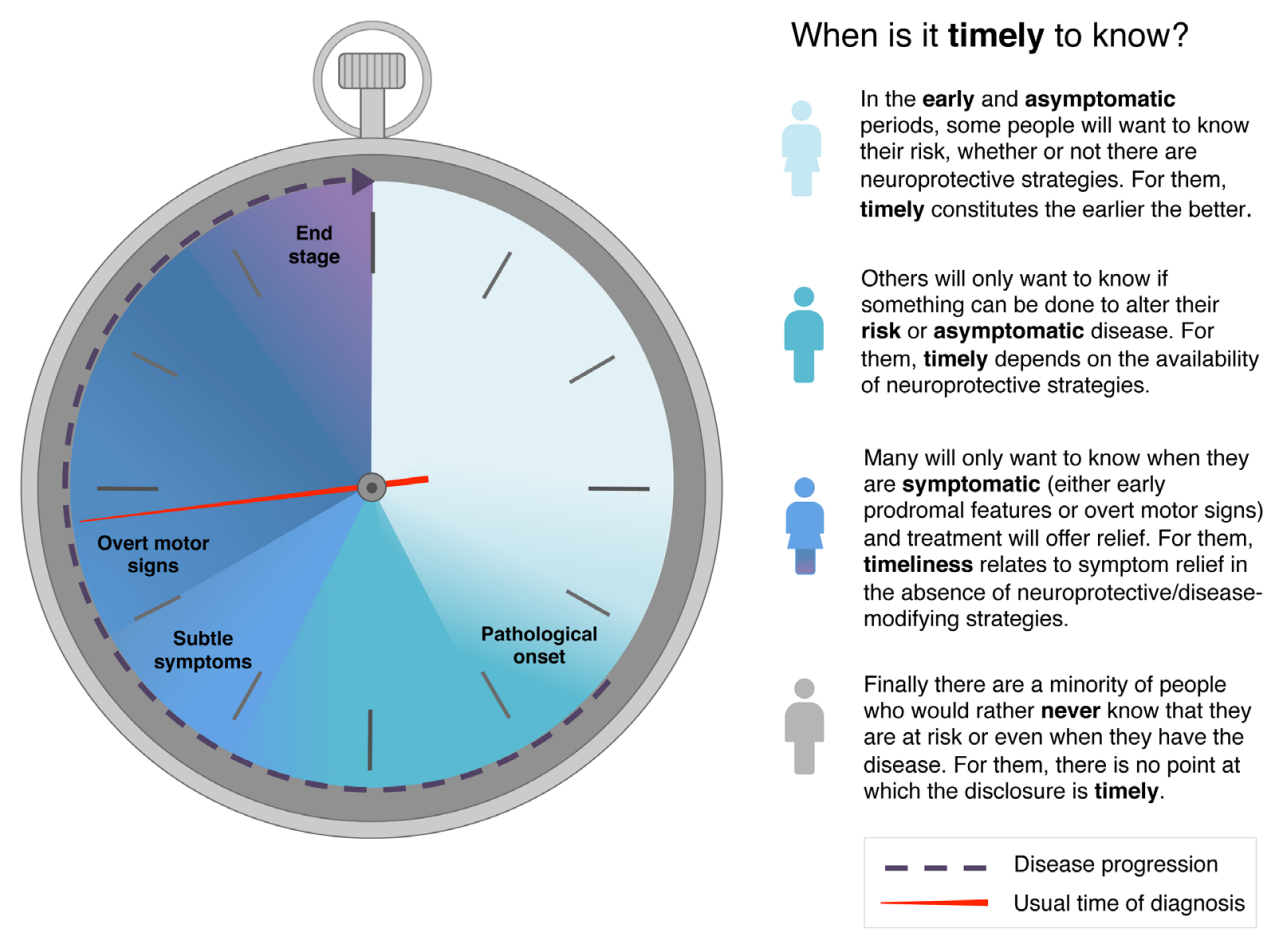
The spectrum of timeliness. Parkinson’s has an insidious onset, and the pathology may start around a decade before diagnosis. Progressive pathology causes subtle motor and non-motor symptoms to gradually accumulate. When the timeliness of diagnosis is considered, a particular individual may fall into one of these four broad categories, depending on multiple factors and personal priorities. As disease-modifying treatments become available, the arguments for moving the “timely” point earlier will become stronger. However, there cannot be a “one-size-fits-all” approach, and shared decision-making and personalized care will determine the optimal point for each person.

Timeliness of diagnosis is likely to depend on a number of personal and societal factors but also on the availability of effective treatments. Personal factors will vary according to the individual’s appetite for knowledge combined with their own weighting of various risk and symptomatic features. This is borne out in the variability of severity seen at first diagnosis of PD: some people seek medical attention at a stage where PD cannot be diagnosed by any current criteria, and some individuals put off seeking medical attention until overcome by a relatively severe burden of disease (for the full range, see Figure 2). In the absence of proven DMT, some may have a personal desire to know as early as possible (for instance, those with a family history or strong personal connection to PD). Others would welcome a diagnosis that explains mild symptoms, while many would continue to be diagnosed in the current way.

The timeliness of a diagnosis of established PD is still a matter of personal perspective, influenced by available treatment options. For many years, delaying anti-parkinsonian treatment was considered desirable in order to delay the onset of treatment complications and because of concerns about levodopa’s toxicity. However, the ELLDOPA (Early vs. Late Levodopa) trial showed that there was no clinical benefit in delaying levodopa and there is also no clinical evidence for levodopa toxicity ^52^. On the other hand, for a newly diagnosed individual, there are both pharmacological and non-pharmacological interventions that increase quality of life. Treatments for the symptoms of PD are well established and effective. Observational studies show that people with PD who remain untreated (by mutual agreement between patient and physician) have worse quality of life across all domains of the PD-specific quality-of-life questionnaire (PDQ-39) when compared with those treated early with anti-parkinsonian medication ^53^. It is plausible to extend this observation to those who are untreated by virtue of the fact that they are undiagnosed but may well be symptomatic. Non-pharmacological management of PD is also evolving. Exercise has been shown to be beneficial for both PD and Alzheimer’s disease (AD) ^54– 56^. Tai Chi and Qigong, dance, and other focused modes of physical activity have been reported to increase quality of life and have positive effects on other aspects of PD ^57, 58^. Furthermore, people with pre-diagnostic features of PD present to their primary care physicians more frequently, often many years before the diagnosis, suggesting that their symptoms are severe enough for them to seek treatment ^10^. As many of these non-motor features are treatable, earlier diagnosis will help identify and treat these early features of PD. These observations all support the rationale of an early diagnosis at a time when symptoms first impact on a patient’s quality of life. This would bring forward the optimal time of diagnosis to the point where motor and non-motor symptoms first present to improve quality of life and long-term outcome of patients with PD.

However, when considering the possibilities, it should be noted that there has been a litany of failed drug trials aiming to alter the underlying disease process. These include nutraceuticals such as co-enzyme Q _10_, green-tea polyphenols, and creatine and pharmaceuticals, including rasagiline, pramipexole, and tocopherol ^59– 61^. Fortunately, the search is not over and is becoming not only more powerful but likely more successful. Several studies are targeting DMTs at particular genetic subgroups, including ambroxol for *GBA* carriers ^40, 62^ and *LRRK2* competitive kinase inhibitors ^63^, and antisense-oligonucleotide trials ^64^ are beginning. The Linked Clinical Trials initiative has identified drugs with proven safety in other areas of medicine, which have sufficient existing data to warrant bringing them to clinical trials ^65^. For example, there appears to be a relationship between diabetes mellitus and PD ^66^, and exenatide is a hypoglycemic agent used for treating diabetes and has had promising results in a small randomized controlled trial in the UK ^67^. A similar story may be emerging around the neuroprotective effects of statins, and a randomized controlled study of high-dose simvastatin is ongoing in the UK ^68^. Two studies that are testing vaccine approaches based on encouraging animal data are under way ^69^. When any interventions are shown to delay onset or progression of PD, it is likely that bringing forward the diagnosis to a time when no treatable symptoms are apparent will become more attractive to individuals with increased risk.

## Benefits beyond those to the individual?

The financial cost of PD is huge, not only for the individual and those close to them but also for society more generally ^70^. In a large study of the economic impact of PD, Kowal *et al*. found that the cost of PD to the US exceeded USD $14 billion in 2010 and that the PD population incurred more than twice as much medical expenditure as an equivalent population without PD ^71^. In the UK, the overall cost of direct health expenditure is around twice that of age-, gender-, and geographically matched controls, and costs increase in line with disease progression ^72, 73^. In AD, economic modeling of DMTs indicates that net savings of as great as £3.3 billion per year are possible ^74^. This suggests that, from a societal point of view, especially given the potential increased prevalence, “timely” diagnosis of PD would be as early as possible when disease-modifying interventions are available.

In addition, there is considerable importance of timely diagnosis to the research field. We have much to learn from recent clinical trials in AD. Unfortunately, all such trials so far have failed to meet their primary endpoints ^75^. Although the reasons for this are not clear and are probably multifactorial, some of the issues are also pertinent to PD research. A recurrent theme of articles reviewing the lack of efficacy of these AD DMTs is that they have all been “too little, too late” ^76, 77^. Similarly, for disease modification in PD to be most effective, it is desirable that initiation be before the majority of the nigrostriatum has been affected (that is, prior to the current point of diagnosis).

## What, then, does a “timely” diagnosis entail?

In line with the approach of personalized care for an established disease, the key to making a “timely” diagnosis is for the clinician to come to a mutual understanding with the patient and incorporate their understanding of the condition (and the likely progress ion of it), their goals, their fears, potential benefits, and possible harm (including medical and psychological) ^78^. The clinician–patient decision-making process weighs up the perceived risks of early diagnosis against the potential benefits, thus maintaining the pillars of medical ethics: autonomy, beneficence, non-maleficence, and justice. Although at present the benefits of early diagnosis for the individual are derived from symptomatic benefit, as more DMT trials are being offered to willing participants, more individuals are likely to define “timely” at an earlier stage.

In conclusion, there cannot be a one-size-fits-all approach to diagnosing PD. “Early” diagnosis exists in a purely temporal and mechanistic spectrum, whereas “timely” diagnosis is tailored to the individual, their priorities, their social milieu, and the therapeutic and health-system options in which they live. In addition to all the quantitative research that will be needed to find neuroprotective treatments, there is a need for robust qualitative research identifying societal attitudes to pre-diagnostic, prodromal, or pre-motor identification of pathology, and a personalized approach to diagnosis, based on the individual’s attitudes, circumstances and available treatments, will be fundamental.
